# Inflammatory Injury to the Neonatal Brain – What Can We Do?

**DOI:** 10.3389/fped.2014.00030

**Published:** 2014-04-09

**Authors:** Noa Ofek-Shlomai, Itai Berger

**Affiliations:** ^1^Department of Neonatology, Hadassah-Hebrew University Medical Center, Jerusalem, Israel; ^2^Pediatric Division, The Neuro-Cognitive Center, Hadassah-Hebrew University Medical Center, Jerusalem, Israel

**Keywords:** inflammation, neonatal, brain injury, neuro-developmental outcome, treatment

## Abstract

Perinatal brain damage is one of the leading causes of life long disability. This damage could be hypoxic–ischemic, inflammatory, or both. This mini-review discusses different interventions aiming at minimizing inflammatory processes in the neonatal brain, both before and after insult. Current options of anti-inflammatory measures for neonates remain quite limited. We describe current anti-inflammatory intervention strategies such as avoiding perinatal infection and inflammation, and reducing exposure to inflammatory processes. We describe the known effects of anti-inflammatory drugs such as steroids, antibiotics, and indomethacin, and the possible anti-inflammatory role of other substances such as IL-1 receptor antagonists, erythropoietin, caffeine, estradiol, insulin-like growth factor, and melatonin as well as endogenous protectors, and genetic regulation of inflammation. If successful, these may decrease mortality and long-term morbidity among term and pre-term infants.

## Introduction

Perinatal brain damage is one of the leading causes of life long disability, cerebral palsy, seizure disorders, sensory impairment, and cognitive limitations (1). The insult may be hypoxic–ischemic, hemorrhagic, inflammatory, or a combination (2). As the evidence for the essential role of inflammation in the pathogenesis of perinatal brain injury accumulates, inflammation is strongly recognized as a major cause leading to long-term injury (3). During inflammation, there is a systemic up-regulation of pro-inflammatory cytokines and diffuse activation of microglia in the neonatal brain (2, 3). Microglia enhance injury by expressing inflammatory mediators and pro-inflammatory cytokines (4). Cytokine-activated cells release toxic substances, such as reactive oxygen species and toxic granules including proteolytic enzymes and myeloperoxidase (5).

The pro-inflammatory cytokines can activate cytotoxic T cells, natural killer cells, lymphokine-activated killer cells, which enhance excessive cellular and tissue damage (5).

This results in cell proliferation, cell differentiation, and cell death, all causing white matter damage (WMD) and long-term neurological injury among pre-term and term neonates (2, 4, 5).

Is it possible to control or reduce this damage?

Since early brain injury has major long-term consequences, efforts should be in limiting this damage in order to reduce morbidity and mortality (1, 2).

Intuitively, if the neonatal immune system is capable of producing an inflammatory response, blockade of inflammatory cytokines may contribute to prevention of early brain damage.

However, the same cytokines responsible for these unfavorable effects in the neonatal brain, as a paradox have been attributed to have beneficial neurotrophic effect (6). Cytokines play a vital role in elimination of cellular debris, and in growth and repair, thus contributing to tissue recovery. Activation of glial cells triggers liberation of factors, such as colony-stimulating factor-1, which are necessary for neuronal survival (6).

This dual effect complicates the task of developing targeted interventions to reduce inflammatory response (6). A better understanding and control of inflammatory processes in the prenatal, perinatal, and postnatal periods may improve the outcome of affected babies.

It is clear that there are other processes, which are beyond the scope of this mini-review that contribute to early neonatal brain injury. Independent and additive to other insults, hypoglycemia for example, which is common in newborns, has been associated with adverse outcomes in term infants, resulting in visual impairment, localization-related epilepsy, and cognitive deficits (7). For the purpose of this review, we have focused on inflammation-related damages to the neonatal brain, and did not discuss other processes.

The objective of this mini-review is to describe current and future perspectives of management strategies designed to limit early inflammatory responses and reduce the risk of early brain injury among term and preterm neonates.

## Avoiding Perinatal Infection and Inflammation

Chorioamnionitis complicates up to 10% of pregnancies, and 35% of preterm labor (8). It stimulates maternal–fetal pro-inflammatory cytokine release, which may interfere with glial cell development and proliferation, at a time in which the brain is most vulnerable. This damage to the developing fetal nervous system increases the risk for adverse long-term neuro-developmental outcome (8).

Therefore strategies based in widespread prenatal education regarding the risks, signs, and symptoms of intra amniotic infections as well as early detection and treatment of maternal infections should be implemented (8, 9).

Timely diagnosis and prompt antibiotic treatment of pregnant women with infection may significantly improve the outcome of both mother and infant (9).

Prenatal group B streptococcus prophylaxis and prenatal measures such as the reduction of nosocomial infections by meticulous hand hygiene, minimizing central line days, and early introduction of enteral feeds may limit inflammatory processes in the newborn infant (9).

## Anti-Inflammatory Treatment

### Corticosteroids

Cortisol has an essential role in the modulation of inflammatory responses. Some preterm infants demonstrate a limited ability to produce cortisol in response to stress (10). Corticosteroids down-regulate inflammatory cytokines, which raises the possibility of cerebral WMD reduction. Should we treat newborn infants with steroids for improving neurological outcome? Currently steroids are recommended antenatally but not immediately after birth (11).

Early dexamethasone treatment (before the first week of life), has been found to be associated with adverse neuro-developmental outcome, and is no longer recommended (12). In a study conducted by Benders et al., ventilated preterm infants were treated with hydrocortisone from 1 week of age onward (13). In this study, ventilator-dependent infants at 1 week of age were considered to be developing chronic lung disease unless a patent ductus arteriosus (PDA) or an infection was demonstrated. No effect was shown regarding brain growth, measured at term equivalent age, after treatment with hydrocortisone for chronic lung disease (13). More data is required before considering early steroidal treatment in the early neonatal period.

### Antibiotics

Although not specifically prescribed for this purpose, certain antibiotics appear to have an anti-inflammatory effect beyond their antibacterial properties (14). Studies have targeted microglial activation as a therapeutic strategy in models of inflammation (15). The tetracycline derivatives, minocycline and doxycycline, which are not routinely used in neonatology have shown to have protective efficacy in cellular injury via microglial inactivation (15). Minocycline reported to variably inhibit microglial activation and to reduce WMD in focal cerebral ischemia in the immature rat brain (15). Lechpammer et al. provide evidence for the protective effect of treatment with minocycline serving as a microglial inactivator when administered over the 96 h time window following early brain insult in a rat model. Most importantly, minocycline appears to have protective efficacy against WMD when administered following the insult. This effect is mediated via reducing the density of microglia (15) through reducing the density of the age-specific target cell – the microglia (15).

Post treatment efficacy has important clinical potential, as brain injury in infants is often detected hours to days after the insult.

Minocycline protection against myelin basic protein loss in WM was concomitant with a decrease in the density of CD-68-positive cells suggesting that minocycline-mediated reduction in microglial cell numbers is a likely mechanism for protection.

However, minocycline may also be acting directly on pre-oligodendrocytes via interruption of oxidative stress pathways, blood–brain barrier (BBB) breakdown, and myelin basic protein degradation (15).

Filipovic et al. studied the mechanism of the neuroprotective effect of minocycline in co-cultures of microglia and neurons from human fetal brain during inflammation induced by lipopolysaccharide (LPS) (16). In neuron/microglial co-cultures, minocycline treatment prevented activation and proliferation of microglia and protected neurons as demonstrated by decreased neuronal cell death. However, the use of tetracyclines, including minocycline, during tooth development has been shown to cause permanent tooth discoloration and enamel hypoplasia, and a decrease in bone growth. Furthermore, emergence of resistant bacteria strains in neonatal intensive care units (NICU’s) is a major issue limiting the use of antibiotics in non-infectious scenarios (15, 16).

### Indomethacin

Indomethacin is a cyclooxygenase inhibiting agent used for the treatment of PDA in preterm infants. Reduction in cerebral, mesenteric and renal blood flow, spontaneous intestinal perforation, as well as platelet dysfunction are recognized as potential adverse effects of indomethacin treatment, and are partially overcome by continuous administration of the drug (17).

Schmidt et al. found that in extremely low birth weight infants, prophylaxis with indomethacin does not improve the rate of survival at 18 months, despite the fact that it reduces the frequency of PDA and severe periventricular and intraventricular hemorrhage (IVH) (18).

However, Miller et al. showed that in newborns born before 28 weeks gestation, the only neonatal factor associated with a reduced risk of developing early white matter injury was prolonged exposure to indomethacin treatment (19).

A randomized trial of prolonged indomethacin treatment is needed to determine whether indomethacin can reduce white matter injury and neuro-developmental morbidity.

## Other Therapeutic Interventions

### IL-1 receptor antagonist

Human neuropathological studies and animal models have revealed IL-1 is implicated in the cascade leading to brain injury at different developmental stages (20). Girard et al. have reported that postnatal administration of IL-1 receptor antagonist in rat pups, who were exposed perinatally to LPS-induced inflammation, preserved both motor function and exploratory behavior. They reported protected stem cell population, prevention of myelin loss in the internal capsule, and less gliosis in treated animals (20). Others have demonstrated that prophylactic administration of IL-1 receptor antagonist to mouse pups, following a perinatal inflammatory insult offered significant protection against bronchopulmonary dysplasia (21).

### Recombinant human erythropoietin

Erythropoietin was shown to have a protective effect against inflammatory injuries in a broad range of tissues and organs (14). A variety of models of neonatal and adult brain injury, highly favor recombinant human erythropoietin (rhEpo) as a novel, effective neuroprotective pharmacological agent (14).

Sifringer et al. report that systemic treatment with rhEpo in a rodent model of brain injury significantly decreased levels of caspase-1-dependent pro-inflammatory cytokines interleukin-1 and 18 (22). Others reported rhEPO treatment coupled to formalin injections, ameliorated neuronal cell death, and normalized the inflammatory response in rats with pain induced inflammatory changes. Treated rats exhibited normal levels of cerebral blood flow, pain sensitivity, and exploratory behavior compared to untreated controls (23).

Liu et al. investigated whether Epo and its derivative carbamylated erythropoietin (cEpo) could provide protection in mouse models of periventricular leukomalacia (PVL) induced by hypoxia–ischemia inflammation. They have found that both Epo and cEpo treatment decreased microglia activation, oligodendrocyte damage, and myelin depletion (24).

The neuroprotective effect of rhEpo has also been shown in experimental rodent models of hypoxia, hypoxia–ischemia, excitotoxicity, and neonatal stroke (25). Moreover, an improvement in long-term neurological outcome following neonatal stroke and hypoxia–ischemia has been reported in Ref. (26). Even in human studies, neurological improvement attributable to erythropoietin was observed at postnatal day 7 in term infants who suffered from moderate to severe perinatal asphyxia who received erythropoietin, compared to control subjects (27). The rates of disability at 18 months of age were also lower in the erythropoietin treated infants (27). Brown et al. found higher mental developmental scores in preterm infants who were treated with a higher dose (400 versus 250 U/kg) of rhEpo for 6 weeks (28).

However, following reports of increased mortality (16% erythropoietin versus 9% placebo) in elderly patients after stroke, there were some concerns regarding safety of high-dose erythropoietin (29). The risk of repeated erythropoietin exposure in adult patients may not apply to the preterm population, for whom the potential benefits may be profound.

One of the possible mechanisms of its neuro-protective effects is an anti-inflammatory effect after binding to its receptor (EPOR), which is expressed on brain cells including astrocytes and microglial cells (30–32).

### Caffeine

Back et al. found reduced cerebral myelination following hypoxia in mice (33). This hypomyelination was related to abnormal oligodendrocyte lineage progression and reduced progenitor pool. Ventriculomegaly was reduced and myelination enhanced in hypoxia-exposed neonatal pups treated with caffeine (33). These observations support the hypothesis that hypoxia inhibits oligodendrocyte maturation and that caffeine administration during early postnatal development may have utility in the prevention of PVL (33).

In a study conducted by Schmidt et al., caffeine therapy for apnea of prematurity improved survival rates without neuro-developmental disability at 18–21 months in infants with very low birth weight (34). However, a recent publication by the same group (35) did not find a statistically significant neuro-developmental advantage at 5 years of age. The “caffeine for apnea of prematurity” trial group found that infants receiving respiratory support appeared to derive more neuro-developmental benefits from caffeine (36).

### Estradiol

In rat pups with oxidative stress, 17 beta-estradiol (E2) showed significant protection against oxygen glucose deprivation induced cell death in primary oligodendrocytes (37). Moreover, E2 attenuated the loss of myelin basic protein labeling in rat pups ipsilateral to carotid ligation. These results suggest a potential role for estrogens in attenuation of hypoxic–ischemic and oxidative injury to developing oligodendrocytes and in the prevention of PVL (37). In a rat pup model, Gerstner et al. showed that E2 produced significant dose-dependent protection against oxygen-induced apoptotic cell death in primary oligodendrocytes (38).

### Insulin-like growth factor

Insulin growth factor-1 (IGF-1) has an important role in brain development and is strongly expressed during recovery after a hypoxic–ischemic injury. Some of its central actions could be mediated through the N-terminal tripeptide fragment of IGF-1: Gly–Pro–Glu (GPE). The neuroprotective properties of local and systemic GPE given after a moderate injury in the developing rat brain were evaluated by Sizonenko et al. who found both local and systemic neuroprotective effect (38). Although the precise mode of action of GPE is unknown, this study suggests that local administration of GPE is neuroprotective after brain injury via modulating glial cells functions through involvement of inflammatory cytokines and antioxidants (38).

Delayed IGF-1 administration rescues oligodendrocyte progenitors (OPS) from glutamate-induced cell death (38, 39).

Wood et al. demonstrate that IGF-1 prevents caspase 3 activation in late OPs when administered up to 16 h following exposure to glutamate (39).

Moreover, late addition of IGF-1 to OPs previously exposed to toxic levels of glutamate promotes oligodendrocyte maturation as measured by myelin basic protein expression. Intraventricularly administered IGF-1 retains OPs in the perinatal white matter after hypoxia–ischemia when given after insult (39). These results suggest that delayed administration of IGF-1 will rescue OPs in the immature white matter and promote myelination following hypoxia–ischemia (39).

Pang et al. tested whether IGF-1 can prevent PVL-like brain damage induced by LPS in the neonatal rat (40). IGF-1 at a low dose significantly prevented LPS-induced deleterious effects without alteration of IL-1beta expression and microglia/astrocytes activation. On the other hand, the low dose of IGF-1 enhanced LPS-induced polymorphonuclear (PMN) recruitment and BBB permeability, and caused intracerebral hemorrhage (40). At higher doses, co-application of IGF-1 with LPS resulted in a high mortality rate. Brains from the surviving rats showed massive PMN infiltration and IVH. However, these adverse effects were not found in rats treated with IGF-1 alone. This study provides the alarming evidence that in an acute inflammatory condition, IGF-1 may have severe, harmful effects on the developing brain (40).

### Melatonin

Melatonin is neuroprotective in adult models of focal cerebral ischemia and attenuates white matter cysts in neonatal mice. Welin et al. found that melatonin attenuates cell death in fetal brain in association with reduced inflammatory response following intrauterine asphyxia in mid-gestation fetal sheep (41). Villapol et al. have demonstrated that melatonin promotes myelination in rat brain model of hypoxia–ischemia by decreasing white matter inflammation after neonatal stroke (42). In a fetal sheep model with umbilical cord occlusion, melatonin had anti-inflammatory effects as it reduced microglial activation (43). The anti-inflammatory effect of melatonin may be mediated by preventing the translocation of Nuclear Factor Kappa B (NF-κB) to the nucleus, thus reducing the up-regulation of pro-inflammatory cytokines (44).

### Endogenous protectors

Neuregulin (NRG-1) is a polypeptide growth factor, which intersects with inflammatory mechanisms. Dammann et al. outlined NRG-1 involvement in perinatal brain damage and suggested it as a potential target for intervention (45). An increasing body of evidence indicates that NRG-1 and its receptors influence the growth and maturation of immature oligodendrocytes (45–47).

Since damage to developing oligodendrocytes is a likely pathogenetic factor in diffuse perinatal WMD, the effects of NRG-1 on developing oligodendrocytes deserve the attention of those who want to prevent WMD and its consequences. In the brain, the neuroprotective effect of NRG-1 exposure prior to middle cerebral artery occlusion is accompanied by a prominent reduction in microglia activation and interleukin-1 mRNA expression, indicating a down-regulation of peri-infarct inflammation by NRG-1 (46). The hypothesis that NRG might have anti-inflammatory and anti-oxidative properties in the brain is further supported by the finding that recombinant human NRG attenuates the production of superoxide and nitrite by stimulated microglial cells (46). Therefore, indirect involvement of the NRG signaling in established protective pathways might be one avenue for the development of improved protection strategies. Thus, NRG-1 might be an endogenous protector in perinatal brain injury (47). NRG-1 might qualify as a potential target for exogenous indirect or even direct neuroprotective intervention (46).

### Genetic regulation of inflammatory processes

Since inflammatory mediators play a dual role in cell death and survival there are trials to reveal the genes, which control these processes. A number of immune-related genes, which are involved in the expression of proteins involved in the inflammatory process, were described. One example is NF-κB. This is a transcription factor that regulates expression of genes involved in inflammation, cell survival, and apoptosis. It enhances brain damage by stimulation of cytokine production. It can be considered as a nuclear component of the cell’s inflammatory response NF-κB inducing kinase (NIK) appears to be one component of the signaling cascade initiated by pro-inflammatory cytokines, such as tumor necrosis factor-α, lymphotoxin-β, and interleukin-1 (47). Nijboer et al. published a study in which they used the NF-κB inhibitor in rats and found that it has a significant neuroprotective effect. In this study, brain damage was reduced by more than 80% with a therapeutic window of at least 6 h and involves down-regulation of apoptotic molecules (48).

Still, much research is required for further understanding of the potential therapeutic role of these results.

### Therapeutic hypothermia

Therapeutic hypothermia in full term infants with a hypoxic–ischemic injury became common and essential practice (49–53). In a recent Cochrane review (52), Jacobs et al. reported that there was evidence from 11 randomized controlled trials (*N* = 1505 infants) that therapeutic hypothermia is beneficial in term and late preterm newborns with hypoxic–ischemic encephalopathy. Cooling reduced mortality without increasing major disability in survivors. The benefits of cooling on survival and neurodevelopment outweigh the short-term adverse effects. They concluded hypothermia should be instituted in term and late preterm infants with moderate-to-severe hypoxic–ischemic encephalopathy if identified before 6 h of age.

## Conclusion

As the evidence for the essential role of inflammation in the pathogenesis of perinatal white matter injury accumulates, examining therapeutic and preventive options is crucial. Although we are far from suggesting that there is one “magic silver bullet” in preventing or treating causes of early brain injury, some of the interventions presented may qualify as potential neuroprotective agents.

The goal is that these interventions will minimize brain injury, thus decreasing mortality and long-term neurological morbidity in term and preterm infants.

## Conflict of Interest Statement

The authors declare that the research was conducted in the absence of any commercial or financial relationships that could be construed as a potential conflict of interest.
